# A suite of ddPCR assays targeting microbial pathogens for improved management of shellfish aquaculture

**DOI:** 10.1128/aem.02149-24

**Published:** 2025-04-02

**Authors:** Mark Ciesielski, Thomas Clerkin, Nicholas Funnell, Tal Ben-Horin, Rachel T. Noble

**Affiliations:** 1 Department of Marine Sciences, Institute of Marine Sciences (IMS), University of North Carolina at Chapel Hill 2331, Morehead City, North Carolina, USA; 2 Department of Veterinary Medicine, North Carolina State University Center for Marine Sciences and Technology (CMAST), North Carolina State University, Morehead City, North Carolina, USA; Indiana University Bloomington, Bloomington, Indiana, USA

**Keywords:** aquaculture, molecular methods, vibrio, pathogens, shellfish

## Abstract

The shellfish aquaculture industry is one of the fastest-growing sectors of global food production, but it is currently facing major challenges stemming from microbial pathogens. This study presents an optimized and validated suite of droplet digital PCR (ddPCR) assays using water samples proximal to oyster farms in North Carolina to quantify pathogens relevant to the aquaculture industry. Two of the molecular assays enable the quantification of the pathogens, *Vibrio parahaemolyticus* and *Perkinsus marinus*, that threaten human health and oyster performance, respectively. This work also introduces two ddPCR assays that enable the simultaneous quantification of at least nine ecologically relevant *Vibrio spp*. using only two sets of primers and probes targeting the glycosyl hydrolase family 18 (GH18) domain of the *chiA* gene in *Vibrio* bacteria. The entire suite of assays was applied to single assessments at 12 sites, revealing heterogeneity in microbial pathogen concentrations across the coastal landscape and variability of abundances within individual estuarine river systems. Additionally, a longitudinal study conducted at a demonstration lease elucidated unique temporal trends for all microbial targets. Notably, when concentrations of *Vibrio spp*. quantified using the two assays targeting the *chiA* gene reached their maximum, the daily probability of mortality increased, suggesting a role for other ecologically pertinent *Vibrio spp*. in the progression of mortality that would otherwise be missed. This study highlights the utility of ddPCR for the advancement of shellfish management by offering insights into the spaciotemporal dynamics of microbial pathogens.

**IMPORTANCE:**

Climate change is drastically altering the environment and changing the abundance and geographical distribution of marine pathogens. These microbial species put additional pressure on the aquaculture industry by acting as sources of disease for animals important to the food industry as well as for humans upon consumption of contaminated food. To address growing concerns, high-resolution monitoring of pathogens can offer insights for effective management in a critical industry. Validated in the field, the suite of molecular droplet digital PCR assays presented here improves upon current methods, enabling the simultaneous quantification of several targets. This technology makes it possible to track pathogens as they move through the environment and reveals changes in abundance that may inform adjustments to farming practices aimed at mitigating negative outcomes. Additionally, this work presents a unique approach to molecular assay design that unveils potential drivers of ecological shifts and emerging etiologies of disease more efficiently.

## INTRODUCTION

The shellfish aquaculture industry is tightly linked with the environment and is constrained by major concerns regarding microbial pathogens that can lead to negative outcomes in animal performance and public health domains (1, 2). Bacteria of the *Vibrio* genus are one such pathogen that is a major concern given that outbreaks in aquaculture settings have the capacity to directly affect the economy on a country-wide scale as well as serve as a threat to public health (3–6). These gram-negative, halophilic bacteria are ubiquitous and naturally occurring in the environment, and with the warming of waters, *Vibrio* bacteria are present for longer periods of time and extend geographical distributions latitudinally (7–10). Shellfish, being filter feeders, are capable of concentrating high levels of *Vibrio spp*. within tissues (11). Consumption of undercooked or raw shellfish that have been colonized by human pathogenic *Vibrio spp*. can therefore lead to gastrointestinal infections with further disease development escalating to septicemia and, in worst cases, death (12, 13). As of now, there have been 12 *Vibrio spp*. that have been implicated as the etiological agent of either wound or gastrointestinal infections in humans, with the leading cause of seafood-related bacterial gastroenteritis in the United States being the species *Vibrio parahaemolyticus* (14, 15). As an effect of climate change, there have also been observed increases in pathogen load within the water column and ultimately, harbored within seafood tissues as a result (16, 17).

In addition to heightened public health concerns resulting from greater abundances and larger geographical distributions of *Vibrio spp*., a number of species within the genus have been implicated as potential etiological agents in mortality events for oysters, one of the most important and fastest-growing sectors of the aquaculture industry (18–22). Although previously published literature debates whether or not a single *Vibrio* species can be considered the primary culprit, the genus is often associated with wide-scale mortality (23–25). Traditionally, culture-based methods have been used to characterize and quantify *Vibrio* bacteria during mortality events; however, these methods lack quantitative power and are limited in their capacity to observe ecological shifts taking place as they happen (26). Additionally, culture-based methods cannot account for the unculturable majority of bacteria present within the environment at any given time (27). As such, assumptions are often made regarding species presence based on bacterial colony characteristics alone (23).

In the aquaculture industry, bacteria are not the only threat to oyster health. Colonization of oyster tissue by the parasitic protozoan, *Perkinsus marinus*, leads to the degradation of tissue, referred to colloquially as Dermo disease, which has put additional pressure on the industry (28, 29). Since 1990, warming waters and elevated salinities have favored the range expansion of *P. marinus* and the intensification of Dermo disease across the Eastern coastline of the United States (30). The transmission of the parasite largely relies on the expulsion of viable pathogens into the water column on a diurnal basis, a process driven primarily by a combination of both higher temperatures and salinity (31). With environmental conditions continuing to change in unexpected ways, it is becoming more difficult to ascertain where and when this protozoan presence will be most prominent. It is also important to consider that the acute stressors from climate change symptoms also impact oyster physiology and condition, making them more susceptible to pathogens and resulting in disease development (32–34).

In the realm of shellfish aquaculture, organismal survival and public health considerations are the two primary concerns from a management perspective (32, 35). Environmental pathogens are a substantial threat to both of these facets of the industry, yet it currently lacks a methodological framework to enable preemptive identification and enumeration of pathogens. Taking lessons learned from the clinical field in response to the most recent pandemic, it is imperative to be poised with effective surveillance technologies and strategies for rapid responses to pathogen presence (36–38). Droplet digital PCR (ddPCR) has been incredibly successful in its application across disciplines, including public health sectors and environmental monitoring, by identifying and quantifying pathogenic genomic material in a variety of matrices (39). Due to the technology’s superior sensitivity across a range of concentrations and rapid turnaround time for results and subsequent analysis, ddPCR offers promising potential for such high-resolution monitoring efforts (40–42). A key advantage to ddPCR technology is its reliance on Poisson statistics for absolute DNA quantification and its inherent dilution effect via partitioning, which enhances resistance to inhibition and grants greater sensitivity, enabling the detection and quantification of targets that exist at low concentrations in complex matrices (43–47). This approach to quantification does not require the generation and use of external standard curves, making it far more reproducible than alternative real-time PCR technologies (48–50).

When considering the sheer number of diverse microbial pathogens that have an impact on the shellfish industry and public health outcomes, the application of ddPCR technologies can improve management by enabling the simultaneous quantification of multiple customizable targets from a single DNA extract. In this body of work, we introduce a combination of ddPCR molecular assays that can provide geospatial and temporal information relevant to all aspects of the shellfish industry. Given the impact that infections from *V. parahaemolyticus* have had on public health, the first assay included in the suite presented here allows for the quantification of the *toxR* gene, a transmembrane regulatory gene, whose primer and probe sequences are specific to *V. parahaemolyticus* (51, 52). Additionally, to address known concerns regarding animal health, this suite introduces a new molecular assay targeting the ITS2 (multicopy internal transcribed spacer region) gene sequence specific to *P. marinus*. Primer and probe sequences for this *P. marinus* sequence target were developed as a derivation of the work first presented by (53) and adapted for ddPCR (53).

To further investigate the dynamics of *Vibrio* bacteria and the role of other species within the genus that could impact the industry, this study also introduces two additional novel molecular assays that allow for the quantification of multiple *Vibrio spp*. using single sets of primers and probes. As members of the *Vibrio* genus are capable of degrading and utilizing chitin as a carbon source, these assays capitalize on the presence of the Chitinase A gene (*chiA*) that enables many *Vibrio spp*. to break down chitin polysaccharides (54–58). The two assays deemed chiA1 and chiA2 (hereafter collectively referred to as ChiA assays) target sequences for distinct regions of the glycosyl hydrolase family 18 (GH18) domain of the *chiA* gene to capture multiple species of the *Vibrio* genus. Engineering the assays to capture multiple species simultaneously ameliorates or completely circumvents the typical difficulties in ascertaining which microorganisms act as etiological agents if they occur at low abundances or inhabit healthy host microbiome structures and become pathogenic later (59, 60). This strategy also takes into consideration that marine diseases can be derived from polymicrobial stressors, with relationships between microbial consortiums being able to transition from benign or mutualistic in nature to antagonistic under a variety of conditions (61–63). Therefore, the simultaneous quantification of several species that comprise such assemblages and have been associated with mortality throughout the industry would be informative to stakeholders and decision-making entities alike.

Together, the simultaneous employment of all the molecular assays described here can make the quantification of human-specific bacterial pathogens, oyster-specific parasitic protozoans, and multiple ecologically relevant bacteria from a single water column sample possible. This is particularly pertinent in the state of North Carolina (NC) given that the oyster aquaculture industry has been actively working towards expansion (64). These efforts have been stymied by increasing occurrences of mortality events that will likely continue to put pressure on the industry with rising disease prevalence exacerbated by persistent environmental stressors (22, 65). A holistic understanding of the inherent complexities of climate change’s impact on microbial dynamics and the fate of the aquaculture industry, as a result, would benefit from the robust data generated from implementing these molecular workflows. This body of work demonstrates the feasibility of using this suite of molecular assays to perform spatiotemporal assessments and has been validated in both field and laboratory settings. The acquired insights from these data can encourage growth and offer support to an incredibly important industry for NC and beyond.

## RESULTS

### Field-based validation of molecular assays

**Fig 1:**
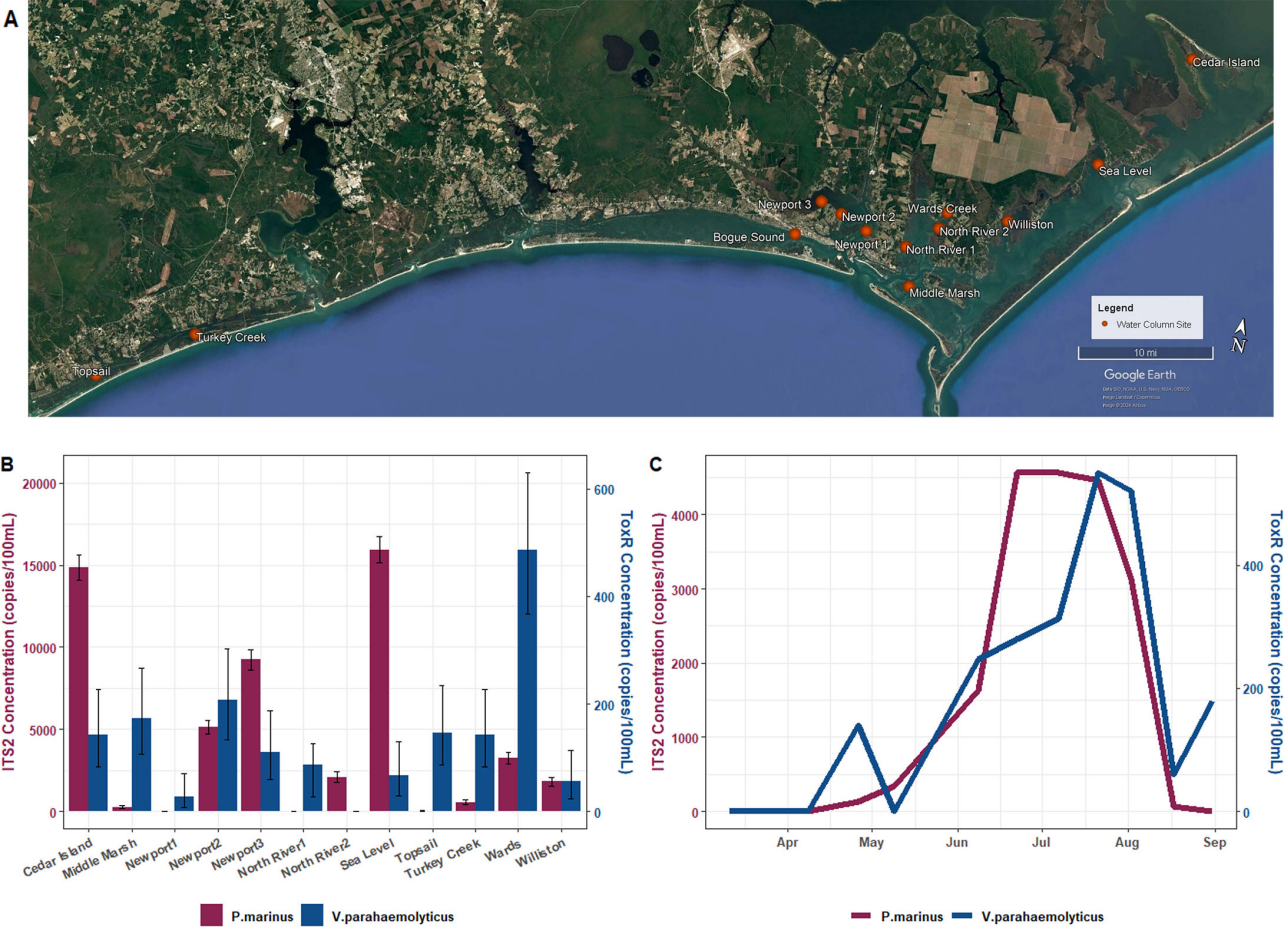
Geographical and temporal assessments of pathogen presence and abundance using ddPCR assays. (A) Two sites across the coast of NC were selected for water sample collection in 2022 due to their proximity to aquaculture operations. The 12 sites spanned a geographical range of ~120 miles and included lease locations that were present within the same water system. (B) ddPCR assays targeting gene sequences specific to *P. marinus* and *V. parahaemolyticus* (ITS2 and *toxR*, respectively) were used to quantify pathogens in the water column across the coastal landscape of NC. Concentrations are reported as gene copies/100 mL of water filtered with Poisson 95% confidence intervals included. (C) A demonstration lease in Bogue Sound, NC was used for a longitudinal study, where water samples were collected on a biweekly basis and analyzed using the molecular assays for *P. marinus* and *V. parahaemolyticus*. Concentrations are also reported as gene copies/100 mL.

Given the level of system diversity across the state, in the summer of 2022, preliminary assessments of method application were conducted at multiple sites proximal to oyster leases (with permission from the lease owners) that have experienced mortality to varying degrees along the NC coastline. Twelve sites spanning ~120 miles of the coast of North Carolina were selected for the quantification of both oyster and human-specific pathogens, *P. marinus* and *V. parahaemolyticus* using molecular assays targeting ITS2 and *toxR*, respectively (Fig. 1A). Single water samples were collected from each site and concentrations of *P. marinus* ranged from below the limit of detection (BLOD) to 15,933.3 copies/100 mL, whereas *V. parahaemolyticus* concentrations fell between the range of BLOD to 486.6 copies/100 mL. *P. marinus* concentrations were highest in water samples from Wards Creek, whereas *V. parahaemolyticus* concentrations were highest in sea level (Fig. 1B). It is important to note that from two of the estuarine systems, the Newport River Estuary and North River Estuary, multiple sites were sampled on the same day to identify intra-system variability. Three oyster lease sites were sampled from the Newport River Estuary, and two sites were sampled from the North River Estuary. From the Newport River Estuary, site 1, closest to the mouth entering the nearby sound, had the lowest concentrations of both *P. marinus* and *V. parahaemolyticus*. Two sites were also sampled out of the North River Estuary on the same day, though both sites had relatively low concentrations of either pathogen.

In addition to the geospatial point measurements taken throughout coastal NC, a longitudinal study was conducted in Bogue Sound due to the site’s recreational value and proximity to ongoing aquaculture operations. A time series at this site revealed that pathogen concentrations varied over time with ranges from BLOD to 4566.6 copies/100 mL of water for the ITS2 gene target and BLOD to 550.0 copies/100 mL of water for the *toxR* gene target. The highest concentrations of *P. marinus* were recorded on 22 June and 7 July. The abundance of *V. parahaemolyticus* began to gradually climb in May before peaking on 21 July, after which concentrations of the *toxR* gene dropped in August. When comparing the timing of pathogen concentrations, both begin to increase in May, but *P. marinus* concentrations reach their maximum 4 weeks earlier than *V. parahaemolyticus*. Though the increase in concentrations for both pathogens differs with respect to timing, the eventual decrease in signal for both targets is most pronounced following the sampling effort on 21 July.

### Novel ChiA assays allow for simultaneous quantification of multiple *Vibrio spp*.

**Fig 2:**
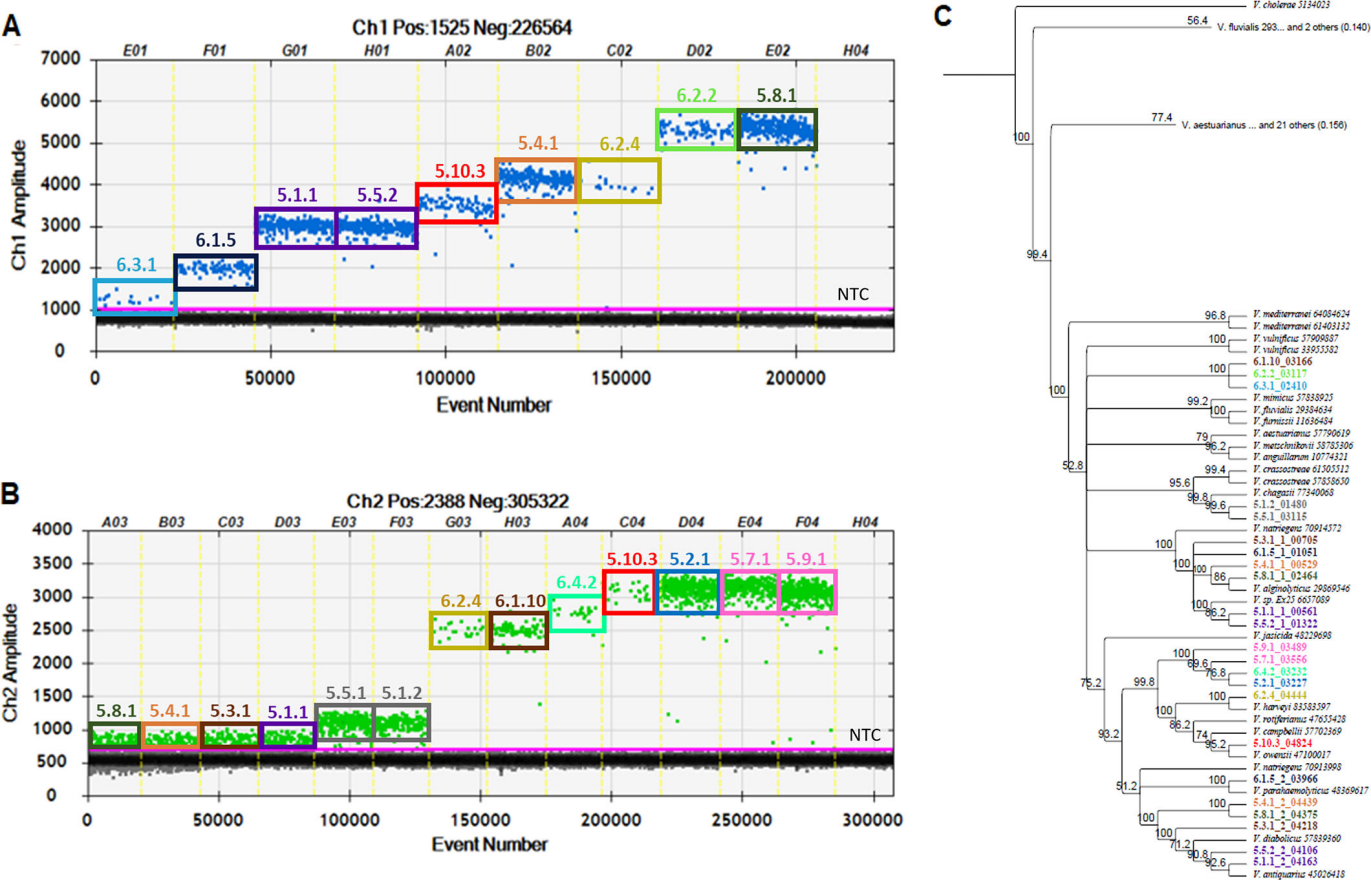
Validation of novel ChiA molecular assays enabling multi-species quantification of *Vibrio* bacteria. Bacteria isolated from oyster tissue on TCBS agar were grown up, extracted, and analyzed using chiA2 (A) and chiA1 (B) molecular assays. Each well in the ddPCR output, separated by yellow dashed lines, contains a single isolate. (C) With RAxML, a phylogenetic tree was generated using hsp60 sequences from isolates and *Vibrio spp*. reference sequences acquired from the NCBI database. Numbers at each node indicate the bootstrap value based on 500 replicates. Two branches of *Vibrio* clades collapsed due to a lack of representation from isolated bacteria, and the tree was constructed using *Vibrio cholerae* as an outgroup with accession numbers following the name of each reference sequence. Colors are associated with individual isolates or groupings and are matched between ddPCR output and the constructed tree.

To determine the effectiveness of the novel chiA1 and chiA2 assays, bacterial isolates originating from homogenized oyster tissue plated on thiosulfate citrate bile salts sucrose (TCBS) agar, preferentially selecting *Vibrio spp*., were screened via ddPCR for the positive signals of either target. Environmental isolates were used to capture the *Vibrio spp*. that are relevant to the geographical systems and have the capability to colonize host organisms that experience mortality on large scales. Analysis of the ddPCR output unveiled differences in the amplitude of fluorescence for droplets that were deemed positive for the respective isolates (Fig. 2A and B). Shifts in the strength of the positive signal suggest that distinct species within the *Vibrio* genus have enough variability in the sequence of the gene target to affect the reaction efficiency and endpoint fluorescence amplitude. Following multi-sequence alignment, subsequent phylogenetic relationship analysis revealed that isolates with distinct fluorescence amplitudes in positive partitions clustered into separate clades (Fig. 2C). This phylogenetic distance between clades further supports the notion that fluorescence amplitude deviations are based on species-specific permutations within the sequences of the *chiA* gene domain. As such, duplexing both chiA1 and chiA2 assays allows for the quantification of multiple *Vibrio spp*. within a single PCR reaction.

**Fig 3:**
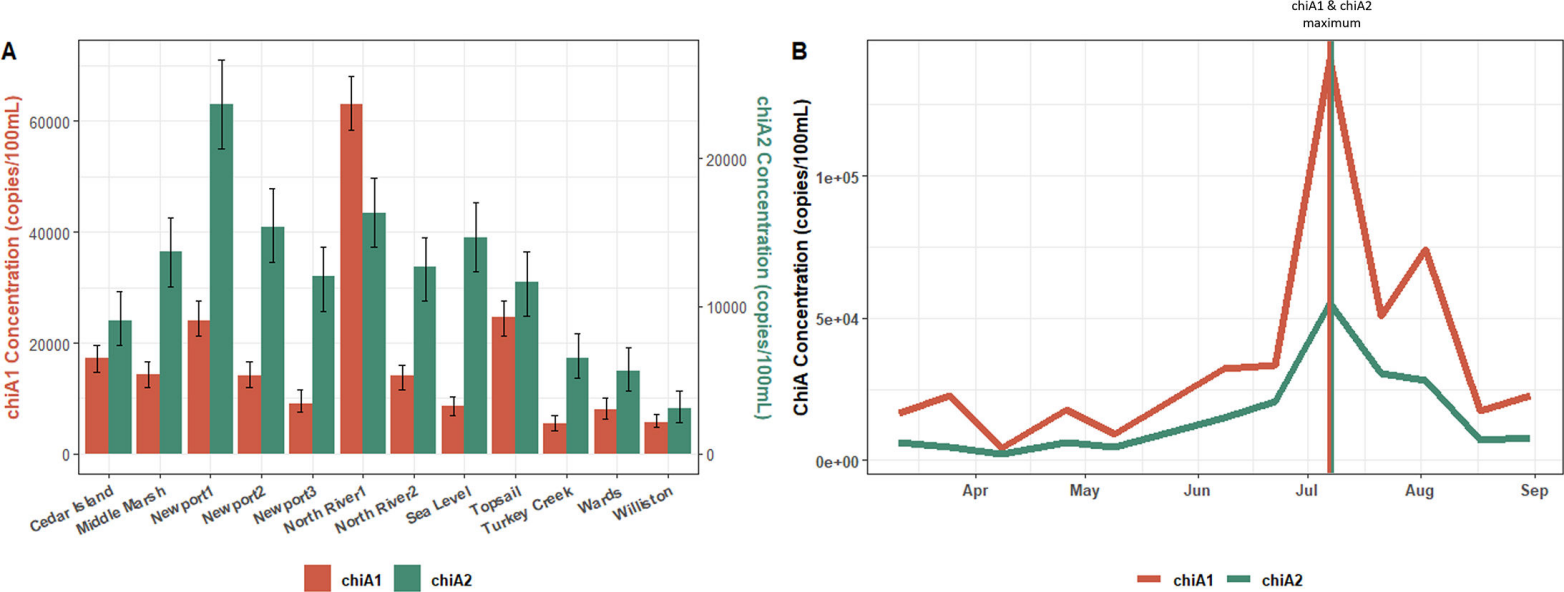
Quantification of *Vibrio* bacteria across space and over time using novel ChiA ddPCR assays. (A) The concentration of chiA1 and chiA2 positive *Vibrio* bacteria in equivalent extracts from the 12 sites across the coastal landscape of NC including Poisson 95% confidence intervals. Concentrations for chiA1 are on the left y-axis with chiA2 concentrations on the right y-axis. (B) Concentrations of *Vibrio* bacteria throughout a longitudinal study conducted out of Bogue Sound, NC. The maximum concentrations of chiA1 and chiA2 positive *Vibrio* bacteria (occurring on 7 July/day 115) are indicated by vertical lines of corresponding colors. Concentrations are reported as gene copies/100 mL of filtered water.

To assess the applicability of these assays for environmental samples, equivalent extracts used for the geographical and temporal analyses described previously were analyzed using both the chiA1 and chiA2 assays to quantify *Vibrio spp*. concentrations. Liberal thresholds were manually set to capture all positive droplets of varying fluorescence amplitude to quantify the total *Vibrio* burden in each sample (Fig. S1). Composite concentrations of chiA1 and chiA2 differed between sample sites, suggesting that differences in *Vibrio*-specific microbial burden and community composition may be the result of unique ecological processes affecting the abundance and diversity of *Vibrio* bacteria at each site (Fig. 3A). When comparing target concentrations from the same sample extract, the highest concentration of *toxR* did not directly translate to high concentrations of either *chiA* gene targets across the sites. In fact, water collected from the North River Estuary closest to the mouth of the proximal sound, designated North River 1, had the highest concentrations of *Vibrio* that were positive for both chiA1 and chiA2 when the same sample had some of the lowest concentrations of both *P. marinus* and *V. parahaemolyticus*. There were also incongruencies in the temporal trends of the larger *Vibrio* genus when compared to the *V. parahaemolyticus* concentrations only. Analysis of ChiA concentrations in Bogue Sound revealed abundance shifts with the highest *Vibrio* burden in the water column occurring on 7 July, before decreasing again (Fig. 3B).

### Application of ChiA assays in relation to oyster mortality

**Fig 4:**
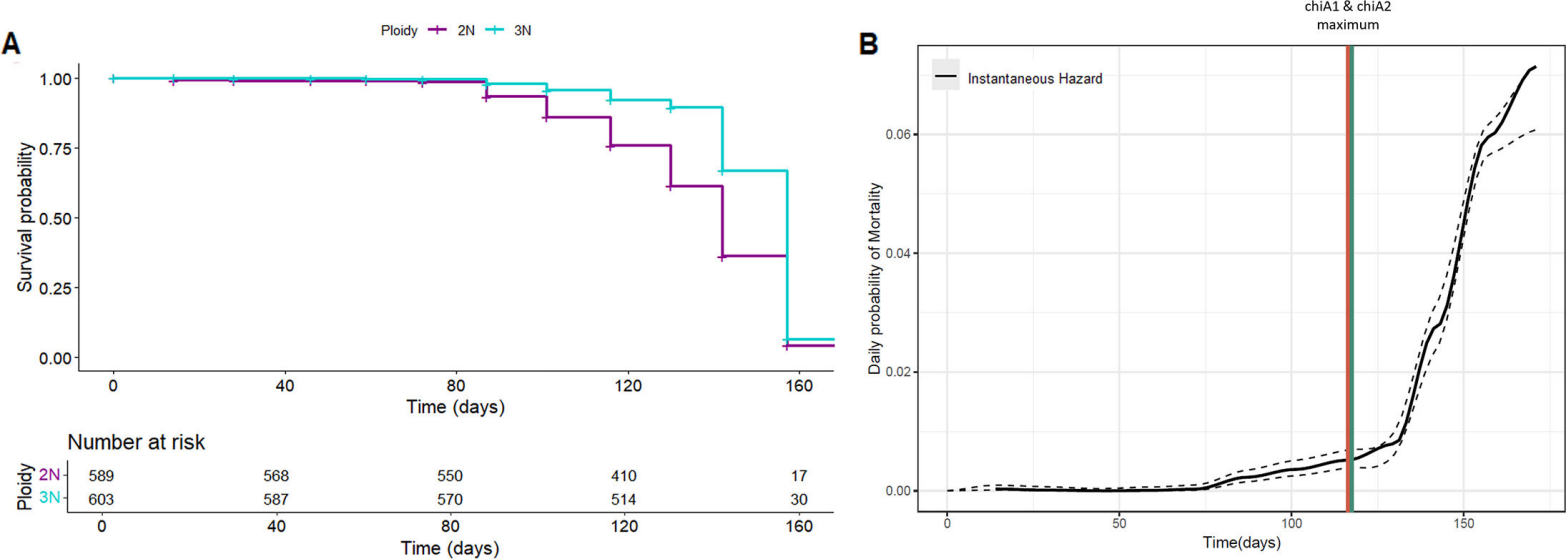
Application of ChiA assays in the context of oyster mortality. (A) A Kaplan-Meier curve was generated to compare survivorship between diploid (2N) and triploid oysters (3N) over a 171-day-long longitudinal study at a demonstration lease based out of Bogue Sound, NC. Oysters were monitored every other week with a subset being censored during each sampling effort. (B) Instantaneous hazard was calculated for each sampling event throughout the study with dashed lines representing 95% confidence intervals. Timepoints for maximum chiA1 and chiA2 concentrations, determined using ddPCR assays, are indicated by vertical lines.

In Bogue Sound, NC, 589 diploid oysters and 603 triploid oysters were deployed in floating bags and monitored every other week to determine whether or not genetic burden would impact survivorship. A Kaplan-Meier survival analysis was conducted to compare survival times (Fig. 4A). There was no mortality until day 72 of the study period, after which mortality was observed during every subsequent sampling effort. The most severe loss, for both diploids and triploids, was observed on day 157 with 27.7% and 54.8% of product found dead, respectively. By day 171 of the longitudinal study, all oysters had expired, concluding the animal monitoring efforts at the lease. Given that all oysters, regardless of genetic composition, did succumb to the mortality event, there was no difference in overall survivorship when considering ploidy in oysters.

To further understand the progression of risk and mortality in the animals, instantaneous hazard was calculated for each sampling effort. The probability of mortality for any given oyster during each sampling event began to dramatically increase on day 130 of the sampling campaign from 0.0079 to 0.014. The instantaneous hazard continued to increase throughout the study period until reaching completion on day 171 when the probability of mortality reached as high as 0.071 (Fig. 4B). Notably, the increase in probability for mortality immediately followed the peak in concentration for both chiA1 and chiA2, suggesting that the increasing presence of *Vibrio* bacteria contributes to the onset and/or progression of an observed mortality event. This particular insight supports the notion that pathogen concentration data derived from novel molecular analysis combined with field-based assessments are able to further our understanding of the temporal progression of disease and, ultimately, animal performance in the oyster aquaculture industry.

## DISCUSSION

This body of work highlights the potential of ddPCR workflows for the acquisition of information critical for the effective management of the shellfish aquaculture industry. In this study, a suite of customized and novel molecular assays was optimized, validated, and incorporated into an environmental monitoring framework. The application of these molecular workflows to quantify microbial species in the water column, even at low abundances, offers a unique perspective on the composition and function of microbial community structure in a variety of environmental systems. This is especially pertinent given that coastal regions, where recreational water activities and shellfish operations are prolific, are particularly susceptible to the effects of climate change (66–68). Challenges such as thermal expansion of water, salinity regime shifts, and excess precipitation can all alter microbial distributions and community compositions, which can negatively impact the health of primary contact recreators and the long-term success of the shellfish industry (25, 69–71). As such, knowledge regarding the spaciotemporal presence and abundance trends of potentially pathogenic species for both marine organisms and human consumers alike is becoming increasingly valuable (11, 72).

### Geospatial variability in pathogen concentrations

Due to their relevance in the aquaculture industry, the suite of molecular assays described here included primer and probe sets targeting gene sequences specific to *V. parahaemolyticus* and included a novel assay aimed at quantifying the parasitic protozoan *P. marinus*. The assays targeting the *V. parahaemolyticus*-specific *toxR* gene sequence and the *P. marinus*-specific ITS2 gene sequence were deemed successful in quantifying genomic material for their respective microbial targets. Positive controls were used to validate the designed assays and presented a clear separation between positive and negative droplets in the QuantaSoft Software ddPCR output. A positive signal in water column samples also indicated that the molecular assays were able to quantify microbial species successfully in complex environmental matrices.

To acquire insights into the geographical distributions of microbial pathogens, water samples were collected from individual oyster lease sites in seven distinct embayments or estuarine systems along the NC coastline. Across the coastal landscape, there were high levels of variability with respect to the concentrations of *V. parahaemolyticus* and *P. marinus*. Samples collected from three sites within the Newport River Estuary alone (spanning ~8 km) contained different concentrations of both microbial pathogens. Of note, large concentrations of *P. marinus* within a given site did not equate to high abundances of *V. parahaemolyticus*, suggesting that the ecology and environmental setting of any given site may favor the presence of oyster-specific parasitic protozoans over human-specific microbial pathogens and vice versa. These apparent site-specific dynamics could prove informative for shellfish management, in that seemingly optimal sites for oyster growth due to a reduced threat from protozoans may harbor higher concentrations of bacteria that in turn present a greater risk to the farmers themselves and/or endpoint consumers. Untangling the environmental and ecological differences that favor one disease source over another will aid in our understanding of the vulnerability to, and protection from, the debilitating diseases that threaten the shellfish industry. With many potential causes poorly understood, the observed lack of correlation between *V. parahaemolyticus* and *P. marinus* concentrations across a dozen sites highlights the necessity of testing for multiple known microbial threats at higher resolutions to fully capture an accurate picture of any farm’s health.

Potential mechanistic factors influencing inter-site variability could include intra-estuarine position, sediment characteristics, flow dynamics, and proximal habitat composition along with the already better-understood drivers of salinity and temperature. Variability in recovered genomic material throughout the workflow should also be taken into account, as heterogeneity in sample composition—even observed within the same system—suggests that generalizations regarding pathogen load should be validated and verified prior to making broad regulatory decisions (Fig. S5). Additionally, increased frequency of sampling throughout longitudinal studies would reveal more accurate relationships between environmental factors and pathogen concentrations over time.

### Unveiling seasonal trends in pathogen abundances

Zooming in on a single lease site, a longitudinal study was also undertaken beginning on 11 March and continued until 31 August to gain perspective on the temporal trends for microbial pathogens in the water column at an isolated location. The quantification of *V. parahaemolyticus* and *P. marinus* using ddPCR indicated that seasonality affected the abundance of each pathogen. Similar to the geographical assessments, there were differences in the trends for both microbial targets. Concentrations of *P. marinus* began to climb and peaked a month earlier than *V. parahaemolyticus* before both saw a dramatic decline in early fall. This corroborates the idea that the presence of one pathogen does not necessitate the proliferation of another, and there are unique responses to ecosystem conditions mandating the assessment of multiple agents of disease simultaneously. It also potentially reveals unexpected dynamics that could play a role in microbial interactions leading to compounding stressors on oyster products.

### Novel ChiA assays reveal larger trends for *Vibrio spp*.

Climate change will also affect the presence and abundance of other *Vibrio spp*. (beyond just *V. parahaemolyticus*) that have either been implicated in oyster mortality events or have the potential to become disease agents, if not already (73). However, designing molecular assays specific to every species has been proven to be both cumbersome and expensive. It is also important to consider that *Vibrio* bacteria are particularly competent, possess multiple genetic exchange mechanisms, and undergo rapid evolution (74, 75). This may impact the specificity of some molecular assays over time as bacteria respond to changing environmental conditions. To combat this, the novel assays targeting two domains of the *chiA* gene introduced in this work were designed in a manner that would capture multiple species within the same PCR reaction. In this way, the resulting ddPCR output allows for more efficient and inclusive quantification of *Vibrio spp*. and a greater understanding of the overall *Vibrio* burden in environmental samples, which would prove critical for regular monitoring purposes.

The geographical assessments conducted for *V. parahaemolyticus* and *P. marinus* were replicated using both ChiA assays (chiA1 and chiA2) to determine differences in the *Vibrio* burden across systems. All samples analyzed were deemed positive for *chiA* signal, consistent with the understood ubiquitous nature of *Vibrio* in estuarine and marine systems (76). As seen with *V. parahaemolyticus* and *P. marinus*, there were prominent variations across the coastal landscape for the general *Vibrio* genus. Curiously, there appeared to be a decoupling between *V. parahaemolyticus* concentrations and concentrations observed for the larger *Vibrio* population quantified using both novel chiA1 and chiA2 molecular assays. For example, both samples collected from the North River Estuary had low or unquantifiable concentrations of *V. parahaemolyticus* while simultaneously having high concentrations of other *Vibrio*. Conversely, at leases where *V. parahaemolyticus* concentrations were highest, i.e., Wards Creek and the second lease in the Newport River Estuary, the overall *Vibrio* presence was less pronounced as indicated by lower concentrations of the *chiA* gene targets. These data indicate that there are unique responses to ecosystem conditions, even within the same genus, and therefore, the application of these novel assays gives us a clearer image of how an assemblage is comprised at any given moment.

Temporal analyses were also replicated by using both ChiA molecular assays on equivalent extracts to elucidate ecological trends for *Vibrio* bacteria over time. The water samples collected over the study period indicated that the overall *Vibrio* burden, for both chiA1 and chiA2 positive species, reached its maximum on 7 July (116 days into the longitudinal study). This peak in concentration of *Vibrio spp*. was observed 2 weeks after the *P. marinus* concentration was at its highest in the water column; again, illustrating that microbial stressors may exhibit unique dynamics and differentially respond to perturbations in the water column. Previous works have also observed that *P. marinus* infections do not correlate with the presence of human-specific *Vibrio sp*. in oyster tissue (77). However, it is possible that the *Vibrio* populations could be acting opportunistically to capitalize on the weakening of proximal host oysters from the apparent presence of the parasitic protozoan and its capacity for immunosuppression (78). It is also worth noting that the *V. parahaemolyticus* concentrations are delayed 2 weeks following the peak in the overall *Vibrio* genus abundance at this lease (Fig. 2C and 4B). This discrepancy indicates that certain species, even from the same taxa, employ unique strategies in the environment and engage in competitive behaviors (79, 80). This temporal analysis, using multiple molecular assays, can provide valuable insights into optimal timeframes for implementing strategies that limit human exposure to virulent pathogens. Additionally, such information can allow for timely farm management adjustments to be made with the goal of reducing the potential of other environmental *Vibrio spp*. from challenging valuable products during periods of high stress.

### Evidence for associations with *Vibrio* bacteria and mortality in oysters

To contextualize this relevance to the oyster aquaculture industry, the survivorship of oysters was assessed on a demonstration lease in NC. Both diploid and triploid oysters were deployed on site to address conflicting reports regarding ploidy-derived differences in animal performance (81, 82). During the longitudinal study, there was a significant mortality event at this lease that compromised 100% of the product, regardless of ploidy, with the last of the oysters expiring 171 days following initial deployment. This was a particularly serious mortality episode given that all oysters succumbed to the mortality event, and genetics alone could not explain the mortality (Fig. 4A). When considering the instantaneous hazard over time, there was a dramatic increase in the probability of mortality following the observed maximum concentration of *Vibrio* present in the water column as determined using the molecular assays for chiA1 and chiA2 (Fig. 4B). These data suggest that an increase in the *Vibrio* concentration within a system may preclude the onset of mortality in oysters and could act as a sentinel signal that would be missed without the application of this novel ddPCR assays. This is consistent with previously published literature that suggests relationships between bacterial burden and oyster tissue damage during oyster mortality events (22). It is also important to note that the acquisition of this data and resulting knowledge does not require the sacrifice of animal products and uses the surrounding water as a representative sample analogous to that of a “liquid biopsy” for a single lease location, providing insights into the presence of biomarkers associated with potential pathogens on a lease in a given moment. Armed with this information and technology, there is a predictive capacity that would encourage preemptive actions and measures to be taken with the goal of mitigating losses rather than having to rely on reactive strategies that are unable to prevent widespread mortality.

### Capitalizing on variable fluorescent signals to interpret individual *Vibrio* species dynamics

There is great utility in understanding species-specific trends given that different clades within bacterial assemblages perform unique biogeochemical and ecological functions. By capitalizing on the variability in fluorescence amplitude across species, the ChiA assays described here are also able to provide granularity at the species level able to distinguish at least nine different taxonomical groups. Multiple radial thresholds can be manually assigned at amplitudes that pertain to individual clades of *Vibrio spp*. within a single well for environmental samples using the QuantaSoft Software (Fig. S2). Concentrations can be calculated for each separate grouping using a subtractive approach and, due to the inherent reliance on Poisson statistics, can be interpreted as individual positive populations to observe temporal fluctuations with finer taxonomical resolution. In this way, these ddPCR workflows can allow for larger trends to be unveiled while also simultaneously identifying if particular *Vibrio sp*. are driving observed outcomes. For example, when applying population selection thresholds to the time series analysis conducted out of Bogue Sound, subpopulations of *Vibrio* can be parsed out, and it becomes clear that certain clades, as indicated by the separate fluorescence groupings of positive droplets, dominate the water column. This finer taxonomical approach shows that the overall *Vibrio* microbial patterns are primarily driven by subpopulations P2, P3, P6, and P7, which, based on previous phylogenetic analysis, are composed of *Vibrio alginolyticus*, *Vibrio owensii*, and other species in the harveyi clade (Fig. 2C, Fig. S3). With this knowledge, research and experimentation can be driven in a more informed manner to explore mechanisms of virulence and disease progression.

### Utility of ddPCR assays and workflows for wide-scale aquaculture management

The potential application of these assays is not limited to oyster aquaculture but would have utility in all molluscan sectors given that a variety of *Vibrio sp*. have been implicated as pathogens for mussels, clams, scallops, and abalone/snails (34, 83). *Vibrio* bacteria have also been associated with disease in finfish and crustaceans and have had negative impacts on the cultivation and farming of economically valuable stocks including salmon, shrimp, lobster, and crab (84–88). With climate change influencing virulence and distributions of *Vibrio* and other problematic microbes, these pathogens act as an even greater threat to the resilience of the aquaculture industry, making these introduced assays incredibly pertinent (2, 89, 90). The ability to quantify multiple species within a single PCR reaction increases clarity in managing risk and facilitates the overall characterization of environmental systems. These molecular workflows also have the flexibility to include additional targets that have proven relevant to individual geographical regions as new etiological agents of disease emerge (91). For example, on the west coast of the United States, OsHV-1 has been challenging oyster products and contributing to mass mortality events (24, 92). Novel assays, in addition to those described in this body of work, could be designed and independently optimized using appropriate controls to track viral and bacterial loads at any given time to assess temporal and geographical risk for aquaculture operations using similar methodologies as described here. The suite of molecular assays would also be useful across multiple facets of the seafood industry, including any stage of production from hatchery to distribution to consumption.

## MATERIALS AND METHODS

### Site selection and sample collection

Single water samples from 12 sites spanning ~120 miles of the NC coastline were collected to capture a snapshot of pathogen load at a given moment. Sites were sampled throughout the months of June and July to capture anticipated windows of higher pathogen proliferation. An additional site located in Bogue Sound, NC was selected to conduct a longitudinal study given the location’s recreational value and proximity to ongoing aquaculture operations. For this time series, water samples were collected every other week to assess temporal variations in microbial presence. This study was undertaken beginning 11 March and continued until 31 August spanning a total of 171 days to best replicate typical farm planting and grow-out industry practices. One sampling effort that was intended to be sampled on 23 May was regrettably missed due to dangerous weather conditions. All water samples were collected adjacent to oyster bags at the surface by boat or, when possible, on foot.

At each site, water was sampled using 1 L polypropylene bottles that have previously been washed with 5% HCl acid solution. Prior to the collection of water, each bottle was rinsed three times with the environmental water at each collection site to ensure a representative sample. Following collection, water samples were immediately placed on wet ice in a cooler before returning to the laboratory for processing. Water samples were on ice for no longer than 3 hours during transport and were immediately processed upon return. Environmental parameters were collected and recorded using a YSI-EXO2 multi-parameter water quality sonde (YSI Inc./Xylem Inc., Yellow Springs, OH) for each sampling effort (Fig. S4).

Additionally, for the longitudinal study conducted in Bogue Sound, diploid and triploid submarket oysters (40–60 mm) from VIMS Aquaculture Genetics and Breeding Technology Center were reared in three replicate mesh plastic floating bags (starting *n* = 200 oysters per bag). Oysters were monitored for survivorship throughout the growing season with single or duplicate oysters being censored during each sampling effort, while dead oysters and residual shells were removed from floating bags. Oysters were determined to be dead if the shells were gaping with no muscular retraction. Oyster monitoring and water sample collection occurred simultaneously.

### Water sample concentration

Each environmental water sample was vigorously agitated by inversion 4–5 times prior to sample concentration to ensure sample homogenization. A total of 150 mL of undiluted water was filtered to dryness through a 47 mm dia., 0.4 µm polycarbonate filter (PC) in triplicate using vacuum filtration manifolds. Sterilized forceps were then used to aseptically transfer the filters to a 1.5 mL microcentrifuge tube (cat no. 3451, Thermoscientific) and stored at −80°C until extraction. Filters were held for no longer than 2 months at −80°C before being extracted.

### Water sample DNA extraction

Environmental DNA was extracted using the MagMAX Microbiome Ultra Nucleic Acid Isolation Kit (cat no. a42357, Applied Biosystems) and with the Purification Automate KingFisher Flex Type 711 (cat No. 5400630). In brief, PC filters were transferred from microcentrifuge tubes to 0.1 mm Glass Powerbead tubes that were provided with the kit followed by an addition of 800 µL of lysis buffer. Each sample was also spiked with 2 µL of cultured halophile cells (*Natronomonas pharonsis*) at a known concentration (3,000 copies/µL template) to calculate the extraction recovery efficiency (Fig. S5) (93, 94). Bead tubes were secured and disrupted using a Mini-Bead-Beater-96 (cat no. 1001, BioSpec, Bartlesville, OK) for 2 minutes at maximum speed. Tubes were then spun down using an Eppendorf Centrifuge 5430 (cat no. 022620596, Eppendorf) at 14,000 g for 2 minutes, and the supernatant was transferred to a Kingfisher deep-well 96 plates (cat no. 95040450, Thermoscientific). Samples were subsequently processed on a Purification Automate KingFisher Flex Type 711 (cat No. 5400630) according to the manufacturer’s instructions with a final elution volume of 100 µL.

### PCR primer and probe design

Lyophilized oligonucleotide primers and fluorogenic probes were obtained from Biosearch Technologies (Novato, CA, USA) shipped salt free and purified via high-performance liquid chromatography, respectively. Probes were labeled either with the FAM (channel 1 in ddPCR output, blue droplets) or HEX fluorophore (channel 2 in ddPCR output, green droplets), and all probes were quenched with non-fluorescent black hole quenchers (BHQ-1).

Primers for the quantification of *toxR* gene sequence specific to *V. parahaemolyticus* were derived from the primers described in reference 51 and adjusted to be compatible with ddPCR. For the quantification of *P. marinus*, multiple sequence alignments of reference ITS2 sequences from *P. marinus* specifically isolated in North Carolina, found in the GenBank database, were performed using the Clustal Omega v1.2.2 algorithm as described previously (53). Primer and probe sequences with the fewest penalties were selected for downstream *in silico* analysis. For the chiA1 and chiA2 molecular assays, a selection of 63 Chitinase A GH18 (glycosyl hydrolase family 18 protein) reference sequences from multiple ecologically relevant *Vibrio spp*. were retrieved from the NCBI database. Sequences were divided into clusters differentiated by gene domain presence, and multi-sequence alignments were performed also using Clustal Omega v1.2.2 embedded into the Geneious Prime software (v2022.1.1,http://www.geneious.com/) to identify regions of unique sequences as a potential target. All resulting primer and probe sequences were checked for specificity using BLAST against the NCBI database (Table 1). For both of the ChiA assays, the assessment of specificity for the capture of multiple species of *Vibrio* was reliant on the top BLAST results for primer and probe sequences based on total score, query cover, *E* value, and percent identity. It was required that the top hits be dominated by members of the *Vibrio* genus. For example, BLAST results for the forward primer of the chiA1 assay reveal that the top 12 results with the highest total score, query cover, percent identity, and lowest *E* value were solely composed of diverse *Vibrio spp*. including *Vibrio crassostreae*, *Vibrio celticus*, *Vibrio pomeroyi*, *Vibrio atlanticus*, etc.

**TABLE 1:** Primer and probe sequences

Gene target	Primer/probe	Sequence	Amplicon size	Reference
ToxR	FWD	GAACCAGAAGCGCCAGTAGT	117	(51)
	REV	AAACAGCAGTACGCAAATCG		
	Probe	FAM-CACAGCAGAAGCCACAGGTGC		
ITS2	FWD	TCAGTCTGGTCGCGAGATTA	96	This study
	REV	AATCTCACACACATCAGGCC		
	Probe	FAM-ACACGCTTGTCGGTTTGCACCATGGC		
ChiA1	FWD	GTTGTAGCCAGAGGTGGTTT	91	This study
	REV	TCCAACAGCACCAAGTATCG		
	Probe	FAM-ACTGCAGGTTGTTGGAACCGTACA		
ChiA2	FWD	CAACCACGGGTGTAGTAAGG	88	This study
	REV	TGAACACTGACTGGGCTTAC		
	Probe	HEX-CCAATGTTGATACGACCTGCTGGC		
GyrA	FWD	ACGATTACCTGCTCTGCTTTAC	120	(95)
	REV	CGTTGAGGTCGAGAACATTGA		
	Probe	HEX-CAAGGGCAGGTCTATCGGCTGAAG		

### Droplet digital PCR

Extracted DNA was quantified using the Qubit 1× dsDNA HS Assays Kit (cat no. Q33230, Invitrogen) with a Qubit analyzer (cat no. Q33226, Invitrogen) according to the manufacturer’s instructions followed by analysis with the ddPCR Supermix for Probes (No dUTP; cat no. 1863024, Bio-Rad). Given that *Vibrio* bacteria are ubiquitous and abundant throughout the environment, all extracts needed to be diluted 1:10 in AE buffer prior to PCR amplification and droplet generation when using the ChiA assays to allow for effective separation between positive and negative partitions in the software output. Dilution of the extracts further benefited the workflow by reducing the concentration of inhibitory molecules that may be present within complex environmental matrices (96–98).

Primer pairs for molecular targets were used in equimolar ratios at 0.9 µM per reaction, and fluorescent probes were used at a concentration of 0.25 µM (Biosearch Technologies). Each reaction mixture contained 5 µL of purified nucleic acids and reached a total volume of 25 µL. In adherence to the MIQE guidelines, each 96-well plate (cat no. 951020389, Eppendorf, Enfield, CT) included a negative extraction control and negative template control in duplicate to verify assay performance and assess contamination (99, 100). Plates also included positive controls for each assay target. For the toxR assay, collaborators provided extracts derived from cultured *V. parahaemolyticus* (NCBI Biosample: SAMN02368309), and for the ITS2 assay, *P. marinus* (Makin et al.) Levine (ATCC 50509) cells were acquired from ATCC and cultured in ATCC Medium 1886 at 25°C. When the cultures reached peak density, cells were centrifuged at 600 × *g* before being resuspended in 800 µL of lysis buffer and extracted using the MagMAX Microbiome Ultra Nucleic Acid Isolation Kit (cat no. a42357, Applied Biosystems) as described previously (see “Water sample DNA extraction,” above). Environmental *Vibrio* isolates were used as positive controls for the ChiA assays and were processed as described in “Bacterial isolate strains,” below. All controls performed as expected, and no contamination was identified during this study. Reaction mixture (20 µL) was used to generate droplets using a Bio-Rad Droplet Generator according to the manufacturer’s instructions, and the resulting emulsion was transferred to a new 96-well plate to undergo PCR amplification using a C1000 Touch Thermal Cycler (Bio-Rad).

**Fig 5:**
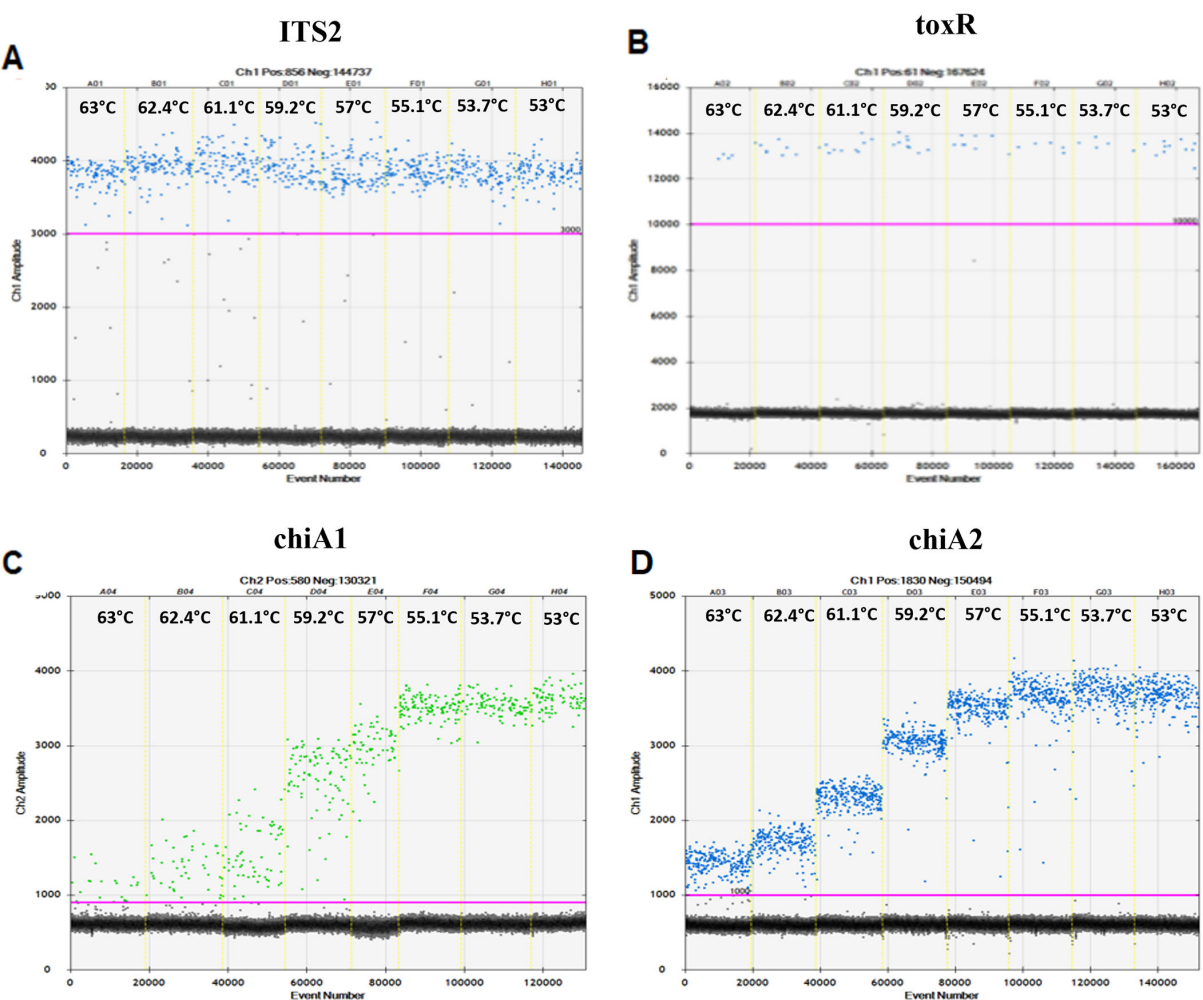
Thermal gradients for the (A) ITS2 assay specific to *P. marinus* using cultured *P. marinus* cells atcc 50509, (B) toxR specific to *V. parahaemolyticus* using cultured *V. parahaemolyticus* cells, (C) chiA1 assay for *Vibrio* target, and (D) chiA2 assay for additional *Vibrio* target both using cultured TCBS isolates. Temperatures ranged from 53°C to 63°C with each temperature labeled at the top of the ddPCR output for corresponding wells. The width of each well is related to the number of final droplets (events) analyzed, with accepted samples needing to have more than 10,000 droplets.

Thermal cycling conditions were selected for each molecular target with the primary step of polymerase activation and template denaturation being performed at 95°C for 10 minutes. After initial denaturation, each assay underwent 40 cycles of denaturation for 30 s at 94°C. Annealing and extension steps lasted for 1 minute with optimal temperatures being determined by conducting thermal gradients from 63°C to 53°C using appropriate positive controls (Fig. 5). Enzyme deactivation was then performed at 98°C for 10 minutes. Plates were then held at 4°C for a minimum of 1 minute prior to droplet analysis. Using a QX200 Droplet Reader (Bio-Rad) and within the QuantaSoft software (*V*. 1.7.4.0917), replicate sample wells were merged, and samples were considered BLOD if there were fewer than three positive partitions and referred to as non-detectable (101, 102). Samples were omitted from the analysis if the average number of partitions was <10,000 accepted droplets (103). Confidence intervals were recorded, and all interpretations of data were based on average values of concentration merged Poisson concentrations between replicates with reported concentrations being transformed from copies per microliter template to copies/100 mL of water filtered as described by reference (104). Due to the coupling of ddPCR systems with software available for analysis, results are presented as QuantaSoft software output figures. For the ChiA assays, *Vibrio* subpopulations were calculated by manually manipulating the threshold within the QuantaSoft software (Fig. S2 and S3).

### Extraction recovery

To assess sources of variability in target quantification for field-based surveillance efforts, a known concentration of *N. pharonsis* cells was spiked into samples prior to extraction and was quantified with ddPCR using the conditions as described in reference 105. Recovery of genomic material was calculated as a percentage with an average extraction recovery of 30.8%. Recovery ranged from 12.96% and reached as high as 41.56% (Fig. S5). Overall pathogen concentrations were not associated with extraction recovery efficiencies.

### Bacterial isolate strains

Visceral tissue of oysters sourced from a commercial farm in North Carolina was homogenized by sonication in a method adapted from reference (77). An initial dilution with 1× phosphate buffered saline (PBS) at an equal volume-to-mass ratio was conducted followed by sonication for 2 minutes with 10 s pauses between each 30 s pulse using a sonicator microtip (model CL-334). Resulting homogenate (100 µL) was transferred to a petri dish with TCBS agar and evenly distributed on the dish with a sterile bacterial cell spreader (106). The plates were allowed to incubate for 20 hours at 26°C, consistent with the temperature of the water at the time in which the oysters were harvested. Colonies were selected individually with sterile inoculation loops and transferred to glass culture tubes containing tryptic soy broth (TSB) supplemented with 1.5% NaCl. Cultures were grown up overnight with constant agitation. Bacterial culture (500 µL) was transferred to 0.1 mm Glass Powerbead tubes, and genomic DNA was extracted using the MagMAX Microbiome Ultra Nucleic Acid Isolation Kit (cat no. a42357, Applied Biosystems) and with the Purification Automate KingFisher Flex Type 711 (cat. No 5400630) as previously described (see section “Water sample DNA extraction”) and was subsequently processed according to the manufacturer’s instructions with a final elution volume of 100 µL.

### Isolate genome sequencing and annotation

To confirm the *chiA* gene presence in *Vibrio* isolates and subsequent assay optimization, extracted DNA was shipped on ice to SeqCenter in Pittsburg, PA, where Illumina sequencing libraries were prepared using the tagmentation- and PCR-based Illumina DNA Prep kit and custom IDT 10 bp unique dual indices with a target insert size of 320 bp. Illumina sequencing was performed on an Illumina NovaSeq 6000 sequencer in one or more multiplexed shared-flow-cell runs, producing 2 × 151 bp paired-end reads. Demultiplexing, quality control, and adapter trimming were performed with bcl-convert1 (v4.1.5). Genomes were assembled using SPADES v3.15.5, and genome annotation was conducted using PROKKA v1.14.5.

### Phylogenetic analysis

Heat shock protein 60 (hsp60 or 60 kDa chaperonin) sequences were selected from the genome of all sequenced isolates and imported into Geneious Prime (v2022.1.1, http://www.geneious.com/). *groEL* gene reference sequences for all characterized *Vibrio spp*. were retrieved from the NCBI database for phylogenetic analysis. Sequences were aligned using Clustal Omega v1.2.2, and phylogenetic trees were generated using a Tamura-Nei genetic distance model with neighbor-joining methods applied and *V. cholerae* (accession no. 5134023) used as an outgroup. Resampling was conducted using a bootstrap method with 500 replicates and a 50% support threshold.

### Survival analysis

Kaplan-Meier curves were generated to present the survival probability of both diploid and triploid oysters during high-resolution monitoring (95). Survival analysis was conducted using the survminer package with instantaneous hazard being calculated using the epiR package in R (107, 108).

## SUPPLEMENTAL MATERIAL

Supplemental figures aem.02149-24-s0001.docx Figures S1 to S5.

ASM does not own the copyrights to Supplemental Material that may be linked to, or accessed through, an article. The authors have granted ASM a non-exclusive, world-wide license to publish the Supplemental Material files. Please contact the corresponding author directly for reuse.
