# Supplementary material for: A suite of ddPCR assays targeting microbial pathogens for improved management of shellfish aquaculture
Source: Appl Environ Microbiol. 2025 Apr 2;91(4):e02149-24. doi: 10.1128/aem.02149-24 (PMC12016556; doi:10.1128/aem.02149-24)
Supplement: Supplemental figures — Figures S1 to S5. [file aem.02149-24-s0001.docx]

**Supplemental Material**

**
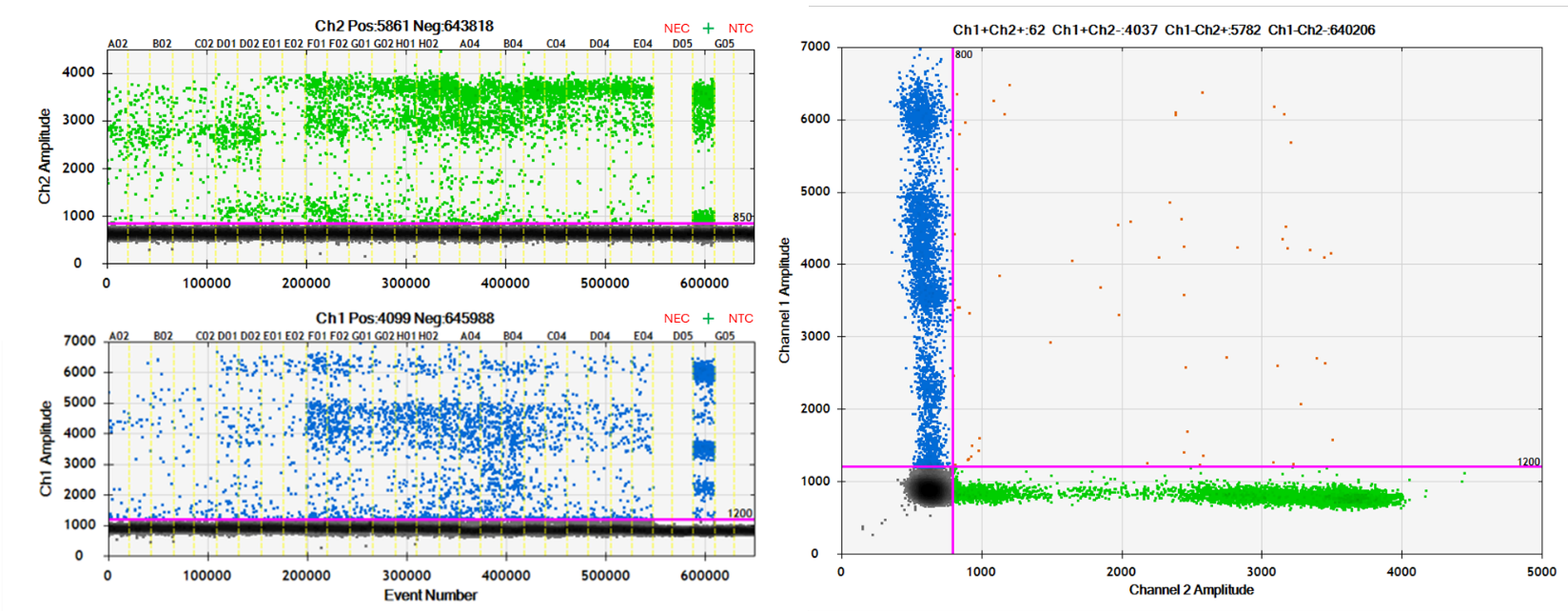
Fig S1** Liberal threshold assignment for chiA1 and chiA2 assays in environmental samples. In the QuantaSoft software, threshold values were manually and arbitrarily set to distinguish between positive and negative droplets. The amplitude of negative droplets was informed by NEC’s, NTC’s, and positive controls that are included with each ddPCR run in accordance with the MIQE guidelines (Huggett et al., 2020). Thresholds were assigned for both channels just above the amplitude of the negative population to capture all positive droplets of varying fluorescence. All positive droplets in individual channels were reported as total chiA1 and chiA2 concentrations.

**Fig S2** Radial threshold assignments and concentration calculations for subpopulations in environmental samples. Using the QuantaSoft software, threshold values were manually set based on positive controls comprised of multiple isolate extracts representative of variable amplitude fluorescence values in a single PCR reaction (Isolate 6.2.2 (P1), 6.2.4 (P2), 5.1.1 (P3), 6.1.5 (P4), and 6.3.1 (P5)). Total concentrations were recorded at each threshold and
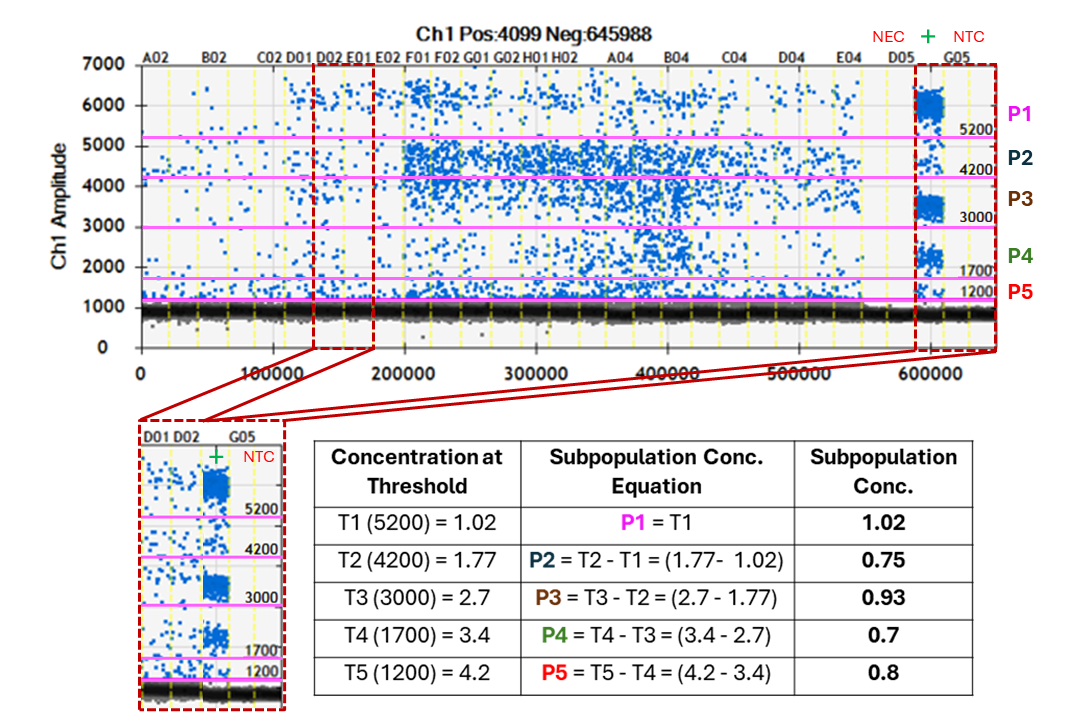
subpopulation concentrations were calculated using a subtractive approach.

**Fig S3** Subpopulation differentiation and quantification throughout longitudinal study. Individual thresholds were manually assigned based on positive control isolate amplitudes to separate output into distinct clades of *Vibrio* for both chiA1 and chiA2 in channel 1 (blue dots) and channel 2 (green dots), respectively. Concentrations for all subpopulations followed in this manner and were interpreted as concentrations of individual clades of *Vibrio spp*. Reported as copies/100mL of water filtered.


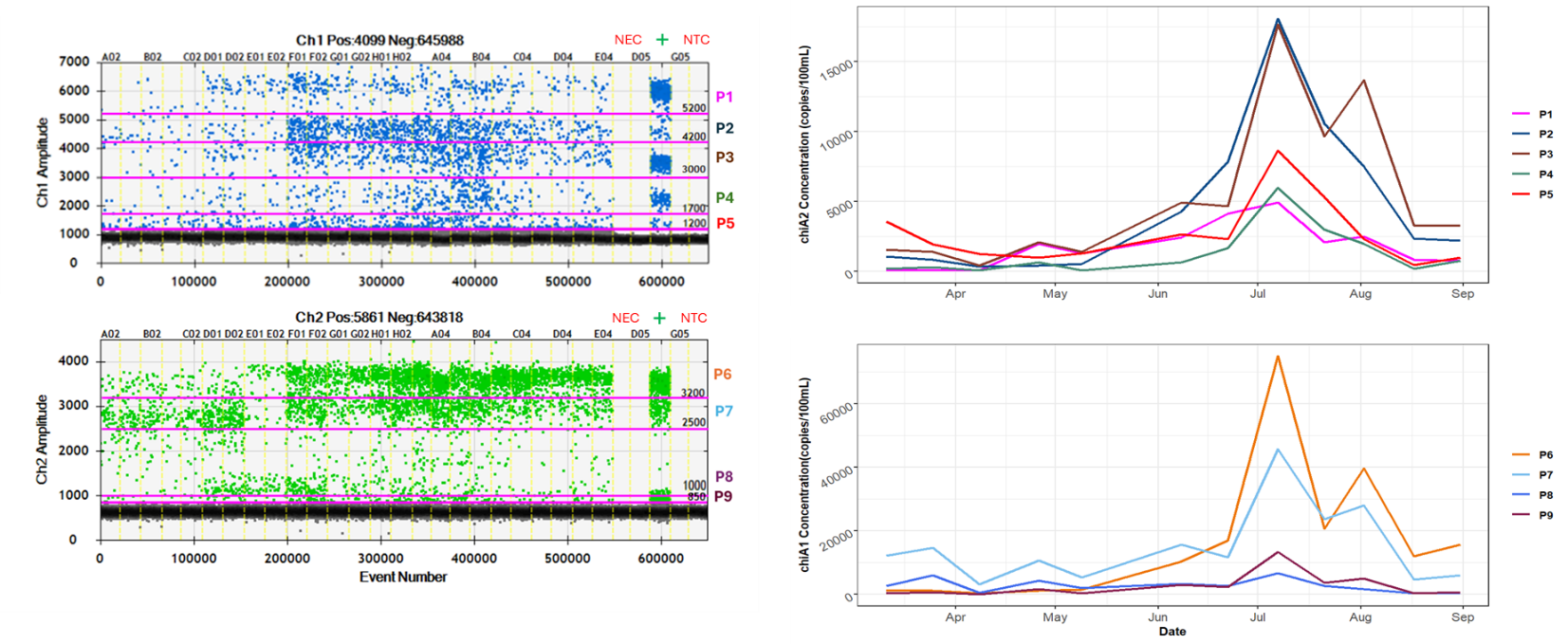


**
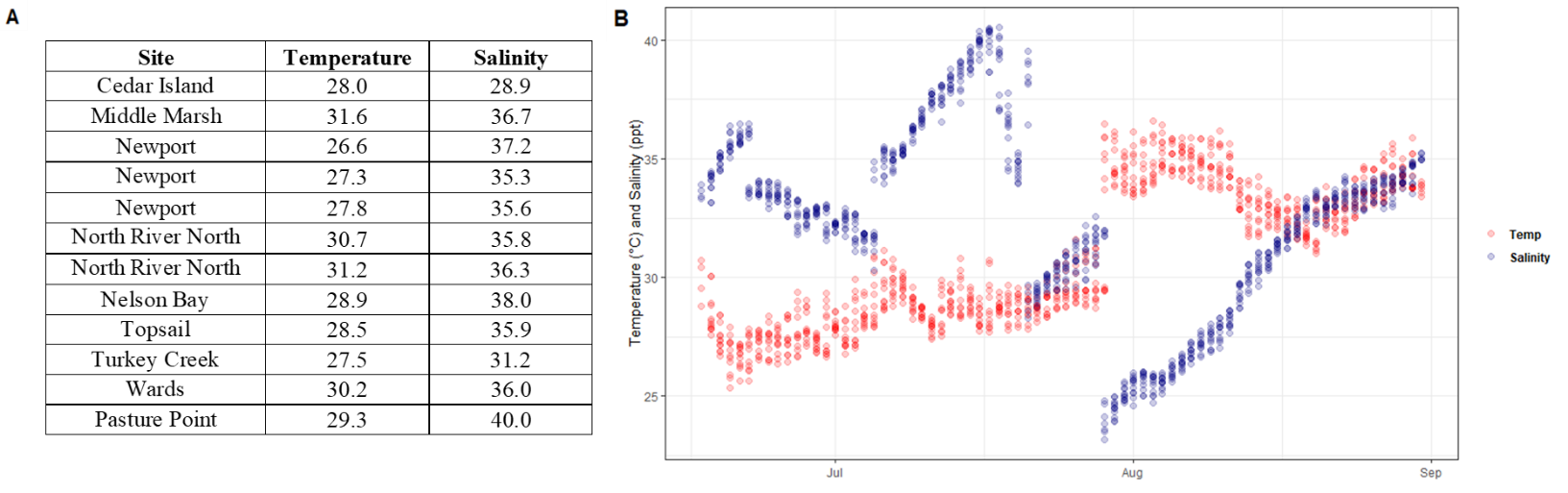
Fig S4** Temperature (°C) and salinity (ppt) measurements from study sites. (A) Point measurements were taken from each site location at the time when water samples were collected using a YSI instrument. (B) A HOBO data logger recording temperature and salinity was deployed for the longitudinal study conducted out of Bogue Sound. Data is shown from 06/17-08/31.


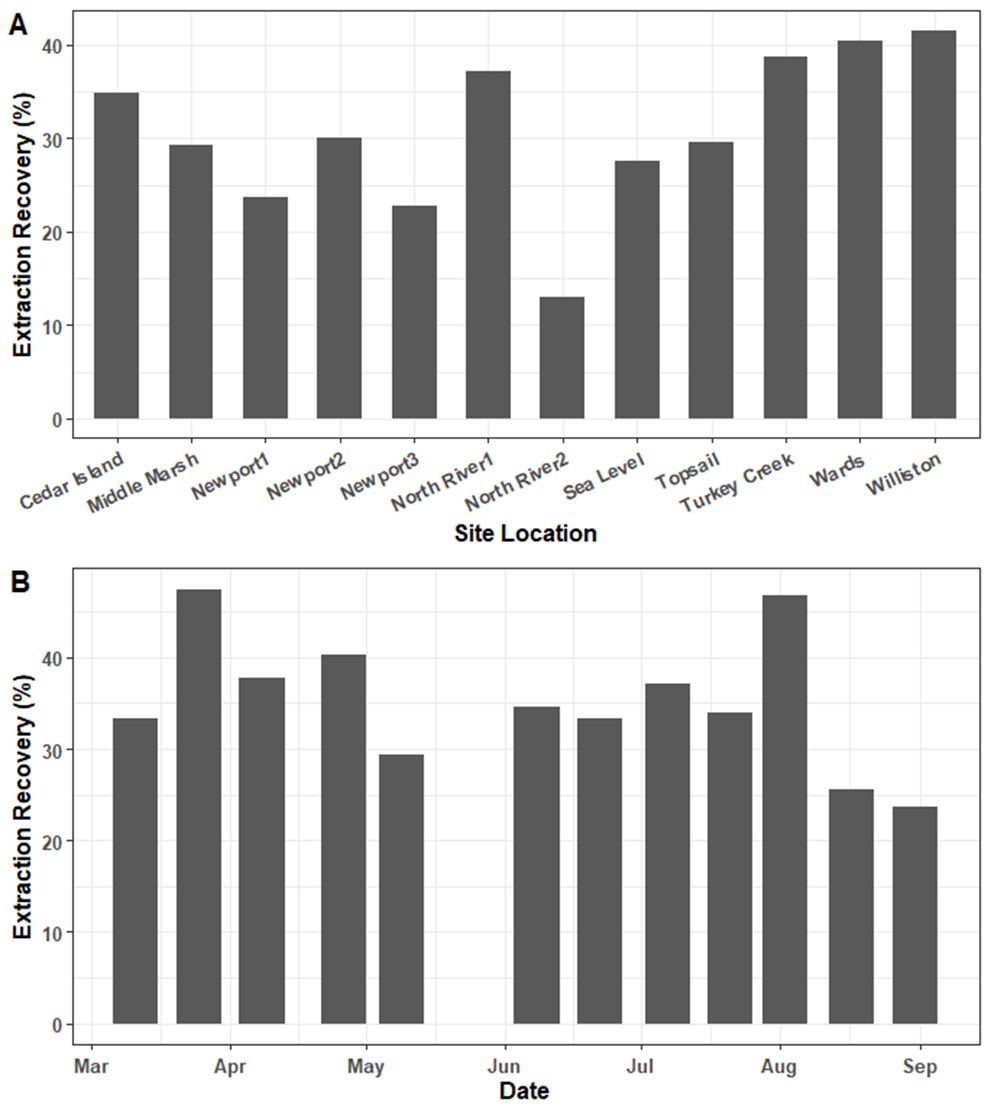
**Fig S5** Extraction recoveries for all samples analyzed using ddPCR. Recoveries were determined by spiking a known concentration of *Natronomonas pharonsis* cells directly into lysis prior to extraction. Using an assay targeting the gyrA gene sequences specific to the cultured cells, ddPCR was used to determine how much genomic material was returned at the completion of the workflow. Recoveries are reported as a percentage. (A) For each sample analyzed, extraction recovery was calculated with the highest recovery reaching 41.6% and the lowest recovery still capturing 13.0% or original spiked genomic material. (B) Extraction recoveries were also calculated for all water samples analyzed for the longitudinal study. Recovery ranged from 23.7% to as high as 46.7%. One sampling effort was missed and no water was collected on 05/23 due to dangerous weather conditions.
